# Draft Genome Sequence of a Metronidazole-Resistant Derivative of *Gardnerella vaginalis* Strain ATCC 14019

**DOI:** 10.1128/genomeA.01345-15

**Published:** 2015-11-12

**Authors:** Jessica A. Schuyler, Eli Mordechai, Martin E. Adelson, Scott E. Gygax, David W. Hilbert

**Affiliations:** Femeris Women’s Health Research Center, Medical Diagnostic Laboratories, a member of Genesis Biotechnology Group, Hamilton, New Jersey, USA

## Abstract

We report the genome sequence of a metronidazole-resistant derivative of *Gardnerella vaginalis* ATCC 14019. This strain was obtained after serial selection to increase the MIC from 4 to ≥500 µg/ml. Two coding changes, in genes encoding a response regulator and an NAD^+^ synthetase, arose during selection.

## GENOME ANNOUNCEMENT

Bacterial vaginosis (BV) affects 29% of women in the United States, making it the most common gynecological infection (1). Although the etiology of BV is polymicrobial (2), *Gardnerella vaginalis* is thought to play a critical role in disease pathogenesis (3). Metronidazole is a recommended front-line therapy for BV (4), and treatment failure is common, affecting roughly 1 in 5 women within 1 month (5). *G. vaginalis* often persists after the completion of metronidazole therapy (6, 7) and resistance has been reported among clinical isolates (8–10). Although further research is necessary to conclusively test the link between metronidazole resistance of *G. vaginalis* and its role in BV treatment failure, it is a plausible hypothesis to explain the poor efficacy of metronidazole in achieving cure.

We serially cultured ATCC 14019 on tryptic soy agar with 5% sheep blood (Northeast Laboratories) containing 1, 8, 16, 32, 64, 128, and 256 µg/ml of metronidazole. Cultures were grown for 48 h at 37°C under anaerobic conditions using the GasPak EZ Pouch system (Becton, Dickinson). Metronidazole MIC values were determined by agar dilution using the same conditions. Genomic DNA was isolated using a Gentra PureGene kit (Qiagen). Genomic DNA was sequenced using the 454-GS Junior system (Roche) following the manufacturer’s instructions. Genome assembly was performed using the GS *de novo* assembler version 3.0. Annotation was performed using the NCBI Prokaryotic Genome Annotation Pipeline (http://www.ncbi.nlm.nih.gov/genome/annotation_prok). Comparative genomic analysis was performed with GS Reference Mapper version 3.0.

We sequenced the genome of ATCC 14019_metR and obtained 122,352 reads totaling 55,721,927 nucleotides. These sequences were assembled into a draft genome consisting of six contigs encompassing 1,660,395 nucleotides, achieving 33-fold coverage. Comparison of this genome to that deposited for ATCC 14019 (NC_014644) revealed 60 high-confidence differences: 5 insertions, 8 deletions, and 47 substitutions. Thirty-nine differences were located in the coding regions of the genome, and we attempted to confirm them by Sanger sequencing. Ten changes were found to be present in our starting stock of ATCC 14019 and likely arose during independent growth from the original clone used for sequencing. Another 27 were not present in ATCC 14019-metR and can be attributed to sequencing errors. The remaining two verified changes were a transversion of guanine to thymine at position 1,662,101, resulting in a missense mutation of lysine (codon AAG) to asparagine (codon AAT) at amino acid position 145 in a hypothetical 280 amino acid protein encoding a response regulator (ADP39446.1) and a transition from guanine to alanine at nucleotide position 786,069, resulting in a change of aspartic acid (codon GAA) to lysine (codon AAA) at amino acid position 376 of NadE, an NH-dependent (3) NAD^+^ synthetase (ADP38714.1). Further study of these genomic changes may provide insight into the molecular mechanism of metronidazole resistance in this species.

### Nucleotide sequence accession numbers.

The whole-genome shotgun project has been deposited in GenBank under the accession number LIYA00000000.
